# Clinical implementation of photon beam flatness measurements to verify beam quality

**DOI:** 10.1120/jacmp.v16i6.5752

**Published:** 2015-11-08

**Authors:** Simon Goodall, Nicholas Harding, Jake Simpson, Louise Alexander, Steve Morgan

**Affiliations:** ^1^ Brighton and Sussex University Hospitals NHS Trust Brighton UK

**Keywords:** quality assurance, beam flatness, photon energy, tissue phantom ratio, beam quality

## Abstract

This work describes the replacement of Tissue Phantom Ratio (TPR) measurements with beam profile flatness measurements to determine photon beam quality during routine quality assurance (QA) measurements. To achieve this, a relationship was derived between the existing ${\text{TPR}}_{15/5}$ energy metric and beam flatness, to provide baseline values and clinically relevant tolerances. The beam quality was varied around two nominal beam energy values for four matched Elekta linear accelerators (linacs) by varying the bending magnet currents and reoptimizing the beam. For each adjusted beam quality the ${\text{TPR}}_{15/5}$ was measured using an ionization chamber and Solid Water phantom. Two metrics of beam flatness were evaluated using two identical commercial ionization chamber arrays. A linear relationship was found between ${\text{TPR}}_{15/5}$ and both metrics of flatness, for both nominal energies and on all linacs. Baseline diagonal flatness ($F_{\text{DN}}$) values were measured to be 103.0% (ranging from 102.5% to 103.8%) for 6 MV and 102.7% (ranging from 102.6% to 102.8%) for 10 MV across all four linacs. Clinically acceptable tolerances of ±2% for 6 MV, and ±2% for 10 MV, were derived to equate to the current ${\text{TPR}}_{15/5}$ clinical tolerance of ±0.5%. Small variations in the baseline diagonal flatness values were observed between ionization chamber arrays; however, the rate of change of ${\text{TPR}}_{15/5}$ with diagonal flatness was found to remain within experimental uncertainty. Measurements of beam flatness were shown to display an increased sensitivity to variations in the beam quality when compared to TPR measurements. This effect is amplified for higher nominal energy photons. The derivation of clinical baselines and associated tolerances has allowed this method to be incorporated into routine QA, streamlining the process whilst also increasing versatility. In addition, the effect of beam adjustment can be observed in real time, allowing increased practicality during corrective and preventive maintenance interventions.

PACS number: 87.56.Fc

## I. INTRODUCTION

Radiotherapy treatments can only be delivered as planned if the radiation beam produced by a linac matches the model used within the treatment planning system (TPS). Routine linac quality assurance (QA) testing is, therefore, recommended to ensure that the produced physical beam properties are maintained within specified tolerances of the baseline values assumed by the TPS.^1^, ^2^ The beam quality, as characterized by its penetrative ability in water, is one such fundamental property. The current standard metric for beam quality measurements is percentage depth dose (PDD) in water. During a commissioning process, a high resolution PDD plot will be captured using a full water phantom.

From this PDD, a tissue phantom ratio (TPR) value may be derived as a beam quality constancy check parameter. This commonly adopted method is often used during routine QA due to its simplicity and practicability, with only dual‐depth, isocentric, ionization chamber measurements being required. At Brighton and Sussex University Hospitals (BSUH), routine measurements are made at 5 cm and 15 cm depth within a Solid Water‐equivalent plastic (WEP) phantom, and a ${\text{TPR}}_{15/5}$ constancy parameter is derived from their ratio, as described in the Materials & Methods section A.

During the production of a clinically useful X‐ray photon beam, a flattening filter is typically used within the linac to produce a beam profile which is flat at a given depth in water. Due to the conical shape of the flattening filter, the energy spectrum of the beam varies across the beam profile with a unique dependence upon the individual filter. If changes arise within the input beam energy spectrum, the interactions with this filter will also vary and modify the flatness of the profile at a given depth. It has been shown that these changes in flatness can be used to monitor changes in beam quality with an increased sensitivity over traditional PDD techniques.^3^, ^4^

At BSUH, two IC PROFILER (Sun Nuclear Corporation (SNC), Melbourne, FL) multi‐axis ion chamber array devices,^(5)^ referred to locally as ICP1 and ICP2, are currently used to monitor the symmetry of photon beams when located within a linac gantry head attachment. If the beam quality could also be validated from the profile flatness data acquired during these measurements, the additional time and equipment required for TPR measurements could be removed, allowing the QA process to be streamlined. In addition, the placement of the ICP within the linac head attachment allows the ICP surface to remain perpendicular to the central beam axis as the gantry rotates. This, in turn, permits an assessment of the beam flatness to be made with ease at any gantry angle or during arc deliveries.

The aim of this work was therefore to establish the relationship between ${\text{TPR}}_{15/5}$ values and beam flatness for the four clinical Elekta linacs at BSUH. From this relationship, baseline flatness values may be derived alongside tolerances which equate to those currently implemented. This will, in turn, allow ${\text{TPR}}_{15/5}$ measurements to be replaced by flatness measurements on a routine basis, streamlining the QA process and improving versatility.

## II. MATERIALS AND METHODS

Measurements were made on four matched linacs: linacs 1–3 (Precise, Elekta Ltd., Crawley, UK) and linac 4 (Synergy, Elekta Ltd.). Linacs 2–4 were dual‐photon energy with 6 and 10 MV photon beams available, whilst linac 1 was single‐photon energy with only a 6 MV photon beam available.

Measurements of ${\text{TPR}}_{15/5}$ were made using a $0.6\;{\text{cm}}^{3}$ ionization chamber positioned within a WEP phantom, and beam flatness was measured using one of the two available ICP devices. Each ICP was calibrated using the recommended wide‐field calibration technique and associated software.^(6)^ The additional steps suggested by Goodall and Morgan^(7)^ were also applied to improve cross‐device calibration consistency.

To capture flatness and ${\text{TPR}}_{15/5}$ measurements for a range of photon beam qualities extending beyond clinically acceptable values, the bending magnet system was progressively adjusted. The bending coarse and bending fine magnets were both altered from the clinical values, maintaining the relative ratio between them and hence the achromatic setup. For each new set of bending magnet currents, the electron gun current servo and automatic frequency control systems were optimized so as to restore a maximum dose rate, and beam steering corrections were made to achieve optimal symmetry. Measurements were then made on each linac of ${\text{TPR}}_{15/5}$, the diagonal and the axial normalized flatness for all variations around the two nominal energies.

### A. ${\text{TPR}}_{15/5}$

Measurements of ${\text{TPR}}_{15/5}$ were made based on the techniques described in the IPSM Code of Practice for high‐energy photon therapy dosimetry.^(8)^ The ionization chamber was placed at the isocenter, defining a constant source to chamber distance of 100 cm, during measurements at depths of 15 cm and 5 cm in WEP. Readings were taken at each depth with a symmetric $10\times 10\;{\text{cm}}^{2}$ field and the ${\text{TPR}}_{15/5}$ was then calculated as

(1) $$TPR_{15/5}=\frac{\bar{R_{15}}}{\bar{R_{5}}}$$

where $\bar{R_{15}}$ and $\bar{R_{5}}$ are the average ionization chamber readings at depths of 15 cm and 5 cm, respectively. The temperature, pressure, and ion recombination corrections were assumed to be equal at both depths.

At our institution, the baseline values for ${\text{TPR}}_{15/5}$, when measured using the technique described above, are 0.703 and 0.752 for 6 and 10 MV beams, respectively. On monthly testing of the ${\text{TPR}}_{15/5}$, a clinical tolerance of ± 0.5% is applied locally to surpass the recommendations of the guidance.^1^, ^2^

### B. Beam flatness

The ICP was positioned with an SSD of 100 cm and a 1 cm slab of Perspex was added to provide buildup in addition to the intrinsic 0.9 cm within the device.^(5)^ The source‐to‐chamber distance was therefore set to 100.9 cm. All measurements were made by integrating the dose of a $30\times 30{\text{cm}}^{2}$ field over a 100 MU exposure. Two definitions of the beam flatness were investigated: the diagonal normalized flatness, $F_{\text{DN}}$, as described by Gao et al.,^(3)^ and the axial normalized flatness, $F_{\text{AN}}$. From the captured profile the beam flatness values were calculated as

(2) $$F_{DN}=\frac{\left(\sum_{i=1}^{4}R_{D}^{i}\right)/4}{R_{CAX}}\;and\;F_{AN}=\frac{\left(\sum_{i=1}^{4}R_{A}^{i}\right)/4}{R_{CAX}}$$

where $R_{D}^{i}$ are the corrected readings from the chambers situated on the positive and negative diagonal axes at ±17.0 cm, $R_{A}^{i}$ are the readings from the chambers situated on the x‐ and y‐axes at ±12.0 cm, and $R_{CAX}$ is the reading from the central beam axis ion chamber. For all readings, the derived chamber specific calibration factors were applied and the background was accounted for.

## III. RESULTS

**Figure 1:**
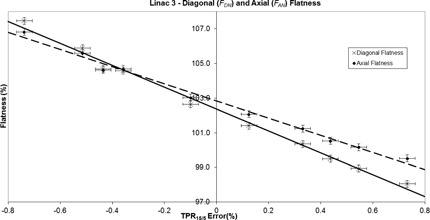
The variation of diagonal normalized flatness (solid line $R^{2}=0.9924$) and axial normalized flatness (dashed line $R^{2}=0.9908$) with ${\text{TPR}}_{15/5}$ error (%) for linac 3 at a nominal energy of 6 MV.

For each linac and nominal energy value, both $F_{\text{DN}}$ and $F_{\text{AN}}$ were found to vary linearly, with the ${\text{TPR}}_{15/5}$ percentage error arising from an investigative range of bending magnet settings. Figure 1 shows the relationship between ${\text{TPR}}_{15/5}$ percentage error and flatness for the measurements performed at 6 MV on linac 3, extending across the clinically acceptable range of beam quality.

When varying the bending magnets settings, a greater response was displayed by the diagonal flatness data ($F_{\text{DN}}$) for every linac and energy combination when compared to the equivalent axial flatness data ($F_{\text{AN}}$). For the 6 MV beams, the mean rate of change in $F_{\text{DN}}$ across all four linacs was calculated to be −4.2%±0.2% per 1% change in the ${\text{TPR}}_{15/5}$ percentage error. For $F_{\text{AN}}$, this value was calculated to be −3.3%±0.2% per 1% change in the ${\text{TPR}}_{15/5}$ percentage error.

**Figure 2:**
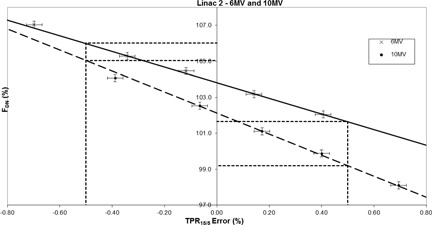
The variation in diagonal normalized flatness with ${\text{TPR}}_{15/5}$ error (%) for nominal energies of 6 MV (solid line $R^{2}=0.9982$) and 10 MV (dashed line $R^{2}=0.9972$) using linac 2. The dotted lines show the ±0.5% clinical tolerance applied locally to the ${\text{TPR}}_{15/5}$ measurements.

Additionally for all linacs, when adjusting the bending magnet current for the 10 MV nominal energy setting, a more rapid change in flatness with ${\text{TPR}}_{15/5}$ percentage error was demonstrated as compared to the 6 MV results given above. For 10 MV, the overall mean rate of change in $F_{\text{DN}}$ across all four linacs was calculated to be −6.1%±0.2% per 1% change ${\text{TPR}}_{15/5}$ percentage error. Figure 2 shows the individual data for linac 2.

Using ICP1, the mean baseline $F_{\text{DN}}$ value was calculated across all four linacs to be 103.0% for a nominal energy of 6 MV with a range of 102.5% to 103.8%. For a nominal energy of 10 MV, the mean $F_{\text{DN}}$ value was calculated to be 102.7% with a range of 102.6% to 102.8%.

When using the ICP2 device, the rates of change of $F_{\text{DN}}$ with ${\text{TPR}}_{15/5}$ percentage error for 6 and 10 MV were found to be equal to those measured using ICP1 within the experimental error of a ±0.2% variation in the rate of change of $F_{\text{DN}}$ with ${\text{TPR}}_{15/5}$ percentage error. Small discrepancies were measured between the baseline $F_{\text{DN}}$ values derived for each ICP which were greater than the $F_{\text{DN}}$ repeatability of ±0.2%. These variations were found to have a mean magnitude of 0.5% and a maximum magnitude of 0.8%.

## IV. DISCUSSION

These results provide similar findings for Elekta Precise and Synergy linacs to those which were shown for a Varian Medical Systems Clinac 2100 by Gao et al.^(3)^ The $F_{\text{DN}}$ was shown to always provide a more sensitive measure of photon beam quality than either $F_{\text{AN}}$ or the traditional measurement of ${\text{TPR}}_{15/5}$. As a result of this work, it has been possible to implement this measurement technique on a routine basis, and streamline the routine and corrective QA processes by maximizing the amount of information which can be captured from a single exposure. In addition, the photon beam quality may easily be assessed with respect to clinically relevant tolerances under more varied conditions, including alternative gantry angles and during arc deliveries.

For the linacs that were investigated, it can be suggested that, when measuring at the 80% width points for a $30\times 30\;{\text{cm}}^{2}$ field, applying $F_{\text{DN}}$ tolerances of ±2% or ±3%, for 6 or 10 MV photons, respectively, would ensure that the ${\text{TPR}}_{15/5}$ was correct within the local tolerance of ±0.5%. It can also be estimated that, if the beam profile maintains less than a ±2% variation from the baseline value, the ${\text{TPR}}_{15/5}$ will remain correct within approximately ±0.25% or ±0.20% for 6 and 10 MV photons, respectively. A device such as the ICP also allows the user to view the beam profile in a real‐time mode. When carrying out corrective action, any changes to the beam quality may therefore be immediately observed and assessed without the need to complete time‐consuming ionization chamber measurements within a phantom.

Although the rate of change of $F_{\text{DN}}$ with ${\text{TPR}}_{15/5}$ percentage error was shown to be comparable between linacs, offsets were measured between the baseline values. These differences may arise from the accuracy with which individual linacs are matched to one another and within the manufacturing process of the linac components, such as the flattening filter. This technique must, therefore, be commissioned for each individual linac and energy combination. Variations in the baseline values were also observed between ICP devices, which may be due to residual uncertainties within the calibration process, despite the additional steps to minimize these.^7^, ^9^ Considerations should therefore be given to establishing separate baselines for each device, or to reduce tolerances, if interchangeability is desired. Using the methods described here, it is suggested that approximately 90 min would be required, per energy, to capture a sufficient quantity of ${\text{TPR}}_{15/5}$ data and corresponding profiles in order to derive the required relationships for two ICP devices.

Once baseline and tolerance values have been established, two key points should be addressed before implementing this technique on a routine basis. Firstly, although the $F_{\text{DN}}$ measurement is largely unaffected by errors within the beam symmetry,^(3)^ it is suggested that a value of ${\text{TPR}}_{15/5}$ is only interpreted from the $F_{\text{DN}}$ value when the beam symmetry is within locally applied tolerances; at BSUH this tolerance is ±2%, based on guidance.^1^, ^2^ Secondly, it is important to ensure the ongoing consistency and reproducibility of the beam profiling device. To ensure this is adequate, an assessment of the accuracy of each profiling device in use should be considered against a full water phantom on an annual basis or following a device recalibration.

## V. CONCLUSIONS

A strong linear correlation was shown between ${\text{TPR}}_{15/5}$ and $F_{\text{AN}}$, and also between ${\text{TPR}}_{15/5}$ and $F_{\text{DN}}$ values for 6 and 10 MV photon beams across multiple Elekta linacs. Measurements of $F_{\text{DN}}$ were shown to have an increased sensitivity to changes in beam quality compared to $F_{\text{AN}}$ which, in turn, was more sensitive than the current constancy check parameter of ${\text{TPR}}_{15/5}$. The use of flatness measurements in place of ${\text{TPR}}_{15/5}$ may, therefore, allow beam quality tolerances to be reduced, whilst simultaneously providing a means to more easily achieve them through direct observation of live beam profiles during corrective action. In addition, flatness measurements have an increased versatility when compared to ${\text{TPR}}_{15/5}$ determination by allowing the beam quality to be validated under varying conditions, such as alternative gantry angles.

Following an initial commissioning time investment of approximately 90 min per nominal energy for two profiling devices, this technique can allow streamlining of the QA process by minimizing the number of exposures and quantity of equipment which is required. The method also presents opportunities for maintaining an improved level of consistency in machine performance due to its enhanced sensitivity and improved practicality, as compared with traditional methods of beam quality determination.
